# Optimizing self-organized study orders: combining refutations and metacognitive prompts improves the use of interleaved practice

**DOI:** 10.1038/s41539-024-00245-7

**Published:** 2024-04-24

**Authors:** Erdem Onan, Felicitas Biwer, Roman Abel, Wisnu Wiradhany, Anique de Bruin

**Affiliations:** 1 School of Health Professions Education, Maastricht University, Maastricht, the Netherlands; 2 Ruhr University Bochum, Bochum, Germany; 3 Atma Jaya Catholic University of Indonesia, Jakarta, Indonesia

**Keywords:** Education, Education

## Abstract

During category learning, students struggle to create an optimal study order: They often study one category at a time (i.e., blocked practice) instead of alternating between different categories (i.e., interleaved practice). Several interventions to improve self-study of categorical learning have been proposed, but these interventions have only been tested in learning tasks where students *did not* create the study order themselves. Instead, they decided which type of study order to follow. This pre-registered experiment examined whether an intervention that combines refutations and metacognitive prompts can enhance students’ engagement in interleaved practice, specifically when they organize the learning materials themselves. Ninety-one undergraduate students were randomized into the intervention and control condition and learned visual categories. Prior to the intervention, students used more blocked practice. After the intervention, the use of interleaved practice significantly increased in both immediate and delayed-transfer tasks. More interleaved practice was associated with better classification performance. Our findings indicate that refutations and metacognitive prompts form a strong intervention that corrects students’ erroneous beliefs and increases their engagement in interleaved practice.

## Introduction

To optimize their learning, students should seek out and engage in *desirable difficulties* as much as possible^1^. Desirable difficulties refer to learning conditions (e.g., learning strategies) that require substantial effort in the beginning of a study session and slow down immediate performance, but enhance long-term learning and transfer^2,3^. For example, when students need to re-study a chapter, they could decide whether to test themselves on the chapter questions or to re-read this chapter. While self-testing requires students to invest more effort into learning than simply re-reading^4,5^, it also leads to better knowledge acquisition and retention^6–8^.

During self-study, however, not all students engage in desirable difficulties^9,10^, indicating that students have difficulties in taking effective control of their learning. For example, one desirably difficult strategy that is scarcely utilized by students is interleaved practice^9,11^. This strategy entails that students introduce variability to their study order by alternating between the exemplars of to-be-learned concepts or categories. In medical school, for example, students learn how to diagnose different types of brain disorders by studying brain images. Interleaved practice requires students to switch between brain images of different disorders (ABCABC) instead of grouping exemplars of the same disorder (AABBCC), called blocked practice. Various studies have encouraged the use of interleaved practice^12–16^ because it has the advantage of fostering mental comparison, stimulating retrieval mechanisms, and preparing students to the unpredicted nature of real-life situations.

Interventions to support self-regulated use of interleaved practice have been proposed in previous studies^17–19^. However, these interventions are mainly tested in experimenter-controlled learning tasks, in which experimenters organize exemplars into blocked or interleaved order, and students make one overall decision to choose which order to follow. Such an overall decision provides a behavioral indicator of students’ willingness to use interleaved practice but bears little resemblance to everyday life, in which students organize learning materials themselves, by making constant decisions about which material to study next^20,21^. Therefore, it remains unclear a) whether the efficacy of previous interventions translates to more authentic situations, where students arrange the order of their learning materials themselves, and b) whether their learning benefits from these self-generated study orders. In this pre-registered experiment, we tested the efficacy of a strategy intervention that combined refutations and metacognitive prompts to support self-regulated use of interleaved practice in immediate and delayed learning tasks, where students created their own study order.

In the desirable difficulties literature, few studies examined how students naturally engage in blocked and interleaved practice^20–23^. For example, Tauber et al.^21^ observed the sequence that students followed in an inductive learning task, wherein students learn broader categories (i.e., bird species) without instructions, only by studying exemplars. Across four experiments, students could choose which next exemplar to study; while the selection format, the number of to-be-learned bird species, and instructions were manipulated. Blocked practice was conceptualized as the number of ‘repetitions’ within a bird family; when the current choice overlapped with the previous choice in terms of the family being studied. Results revealed that students largely blocked their learning; on average, the use of interleaved practice never exceeded 30%.

Contrary to Tauber et al., Kornell and Vaughn^22^ found a more nuanced picture. Again, students learned visual categories (i.e., penguin species) by studying exemplar images. Results revealed that students blocked 47% of the trials, which was significantly higher than chance level (15%) but was lower than the blocking rate observed by Tauber et al. (70%). Nonetheless, using a different methodology, Yan et al.^23^ provided further support for students’ preference for blocked practice. In two experiments, students were asked to imagine they were learning painting styles, and they should organize the exemplars in a way that would represent their actual study sequence. These sequences were then qualitatively classified based on the maximum run length of blocked trials (e.g., purely blocking, purely interleaving, blocking to interleaving, etc.). Results revealed that most students would purely (83% in Exp. 3) or largely (~70% in Exp. 4) block to-be-learned painting styles.

Do students prefer blocked practice in other domains than perceptual category learning? Addressing this question, Hartwig et al.^24^ examined how students organized lessons and practice problems of an imaginary math class, while adopting the viewpoint of a teacher who aims to maximize students’ learning. In Exp. 1, students on average, blocked 71% of the practice problems, while 29% of the problems were interleaved. Note that students interleaved the practice problems mainly on the last day before a hypothetical exam (43% of the interleaved problems). Accordingly, researchers manipulated the timing of a surprise exam (at week 3 and 7) and introduced students to different types of scheduling that incorporates some form of interleaved practice (e.g., none, on the last day, throughout, etc.). The findings revealed that students still predominantly blocked the practice problems (76% and 61% for a surprise test at Week 3 and 7, respectively) – despite showing some knowledge about the effectiveness of interleaved practice. Together, these findings indicate that students have a strong preference for blocked practice, even in situations where they are not directly involved in actual studying – thus, no learning effort is required – but only prepare study schedules. Importantly, this preference seems to remain consistent across various learning domains.

The ‘Study Smart’ framework^25^ and the ‘Knowledge, Beliefs, Commitment, and Planning’ (KBCP) framework^26^ are two recent frameworks that explain how students can be trained to use desirable difficulties in a self-regulated manner. Both frameworks concur that it is necessary for students to develop accurate knowledge about different study techniques and to believe that effective study techniques would work for them. However, several factors might prevent students from meeting these preconditions. At the cognitive level, students may have misconceptions regarding the efficacy of blocked and interleaved practice, such that students often believe blocked practice leads to better learning than interleaved practice^9,27,28^. If not corrected, such misconceptions might prevent students from developing accurate knowledge about learning strategies.

At the metacognitive level, students may be misled by their on-task experiences, which result from their engagement in effective study techniques^5,17^. Note that effective study techniques are effort demanding, and this effort investment pays off largely in the long-term. Thus, it is natural that students experience high effort and low learning during initial study. Yet, if students are not aware of these misleading immediate experiences, they might conclude that effective study techniques do not work for them^1^ (‘As I struggle a lot, I am not learning well; thus, this strategy does not work for me’), resulting in a triangle between perceived effort, perceived learning, and study decisions, known as misinterpretation (or inaccurate monitoring) of effort^5,29^. It is plausible that students’ decision to engage in desirable difficulties, and interleaved practice, is influenced by the level of support they receive both at the cognitive and metacognitive level.

A small but growing body of research has aimed to promote the use of interleaved practice in *experimenter-controlled* learning tasks, in which students did not organize learning materials themselves but indicated their preferred strategy^17–19^. This research tested the efficacy of different instructional techniques, including theory-based minimal instructions, experience-based metacognitive prompts, performance feedback, or their combinations. In a recent study, Sun et al.^18^ provided students with minimal instructions and performance feedback. Students learned painting styles, using blocked and interleaved practice, and took part in a classification test. Afterwards, they reviewed their test scores and were informed that interleaved practice leads to better learning than blocked practice. After this intervention, about 60% of students chose interleaved practice to study novel materials, even after a short delay (two days).

Recently, we advocated for an approach that combined theory- and experience-based methods, wherein we also proposed several changes^30^. Given that students often have persistent erroneous beliefs about their study routines, we proposed that refutations may be a promising type of theory-based support to change their beliefs and study behavior. Unlike standard instructions, refutations do not present normative information alone but also explicitly challenge erroneous beliefs^31^. This is achieved by directing attention toward such beliefs (e.g., humans use only 10% of their brain), highlighting their inaccuracy (e.g., this statement is false), and offering a scientifically grounded explanation (e.g., the human brain remains active at all times; no brain region has complete inactivity in terms of blood flow). This structure helps learners to co-activate both correct and incorrect information^32^, thereby stimulating cognitive processes that are essential for conceptual change, such as cognitive conflict^33^ and dissatisfaction with existing mental models^34^, especially when scientific explanation is intelligible and perceived plausible by learners^35^. Research has demonstrated that refutations can successfully counter neuro-myths (e.g., learning styles), scientific misconceptions (e.g., heavier objects fall faster) and misinformation (e.g., vaccines cause autism).

Regarding the content of refutations, we further recommended that refutations warn students about potentially misleading on-task experiences *and therefore precede* the study phase. Note that effective strategies are often associated with high effort and low learning during initial study, while suboptimal strategies might feel easier and give an illusion of learning. An important advantage of providing refutations before the study phase is that students gain a heightened awareness of the challenges they might face during the study phase. In contrast, if theory-based support overlooks these characteristics, and takes place after the study phase, students may struggle to overrule their own experiences, as these experiences will likely support their erroneous beliefs, while contradicting normative information. In this context, refutations act as a pre-exposure warning, known to reduce reliance on erroneous beliefs^36^.

Nonetheless, any theory-based information may end up in vain if not supported by personal experiences; students might consider themselves an exception to a general rule (e.g., this strategy may work for others but not for me). In our view, this challenge necessitates the practice opportunities with learning strategies and experience-based support. To this end, we suggested that students can be provided with visual metacognitive prompts during strategy implementation. These metacognitive would help students to zoom out of their immediate on-task experiences and focus on temporal developments, through which students can recognize the long-term benefits of interleaved practice and verify the content of refutations. Our findings revealed that combining refutations and metacognitive prompts significantly improved the use of interleaved practice in a delayed (one week) near-transfer learning task: 88% of the students chose interleaved practice to study bird-species, but refutations appeared to be necessary and sufficient condition for improvement.

Albeit indirectly, several studies examined whether students increased their use of interleaved practice in self-organized study schedules^24,37,38^. These studies revealed that students showed modest improvements in their use of interleaved practice depending on learning goals^37^ and task characteristics, such as category similarity^38^. For instance, Abel examined the role of motivation (i.e., learning goals), the extent to which students are motivated to tell apart to-be-learned categories. Students learned different types of mushrooms, and in the high-motivation condition, mushrooms were presented under two superordinate categories labeled as edible or poisonous. In the low-motivation condition, superordinate categories were labeled as growing on acidic or neutral soil. Interleaved practice was captured in the ‘switch’ decisions from one superordinate category to another, and it was argued that students in the high-motivation condition would interleave more frequently than students in the low-motivation condition, due to the salient consequence of confusing. The results supported this expectation, but importantly students still largely blocked their learning materials: The rate of interleaving was 20% in the high-motivation conditions, while it was 12% in the low-motivation condition.

Aside from these indirect manipulations, previous studies have not specifically focused on enhancing the use of interleaved practice in self-organized study schedules directly via strategy interventions. This gap is of paramount importance because experimenter-controlled learning tasks tend to oversimplify the complexity of authentic situations, in which students usually make item-level decisions (e.g., whether to re-study a category or which category to study next). Arguably, item-level decisions may overwhelm students, by imposing secondary load^39^ (i.e., cognitive load that results from self-regulatory processes), and hinder their engagement with interleaved practice. Equally important is the uncertainty surrounding the true benefits of strategy interventions on learning outcomes. Earlier investigations into interleaved practice within experimenter-controlled learning tasks yielded a strong benefit of interleaved practice^17,18^. However, the results are mixed when students organized learning materials themselves: Some studies reported a positive correlation between the classification accuracy and interleaved practice (i.e., frequency of switch decisions)^37,38^; while, other studies found no such correlation (Exp. 1)^38^. Together, these limitations call for a comprehensive investigation into the utilization of interleaved practice and its educational advantages within learning scenarios that resemble real-life situations.

In summary, several factors might prevent students from using interleaved practice. Although prior strategy interventions provided valuable insight into how students can be trained to overcome these obstacles, those interventions are limited to experimenter-controlled learning tasks. In authentic learning, however, students need to create their own study order and make multiple decisions in one study session. Therefore, it is crucial to expand on these previous findings, by testing a) whether students can be supported to use interleaved practice in learning tasks, where they organize learning materials themselves, and (b) whether they benefit from these self-generated orders in terms of learning.

The present study aims to improve the self-regulated use of interleaved practice through a strategy intervention that combines refutation-based instructions (henceforth, refutations) and experience-based metacognitive prompts (henceforth, metacognitive prompts) in a learning task, in which students create their own study order. In this paradigm, students learned visual categories (i.e., painting styles and bird species) in an order they created themselves, by making item-based choices. Half of the students were provided with the intervention. The refutations challenged their erroneous beliefs about blocked and interleaved practice and warned them about misleading on-task experiences. The visual prompts showed them how their on-task experiences changed across time and varied between learning strategies. We examined the influence of this intervention on learning strategy beliefs (RQ1) and self-regulated use of interleaved practice (RQ2) in immediate and delayed-transfer learning tasks. Because the efficacy of interleaved practice in this free-choice paradigm is inconclusive^20,38^, we also examined the influence of interleaved practice on category learning (RQ3).

Regarding RQ1, we expected that perceived effectiveness of blocked practice would be larger than of interleaved practice before the intervention. Across time, we expected that perceived effectiveness of interleaved practice would increase, and this increase would be larger in the intervention condition than in the control condition. Regarding RQ2, we expected that pre-intervention use of blocked practice would be more frequent than of interleaved practice. Across time, we expected an increase in the use of interleaved practice, and this increase would be larger in the intervention condition than in the control condition. Finally, regarding RQ3, we expected interleaved practice to benefit category learning: The more students engaged in inter-category switches, the higher their classification accuracy.

## Results

### RQ1. Learning strategy beliefs across time and between conditions

We examined participants’ pre-existing beliefs about blocked and interleaved practice, using a paired sample t-test. The results revealed that perceived effectiveness of blocked practice (*M* = 4.39, *SD* = 0.85) was higher than of interleaved practice (*M* = 4.00, *SD* = 1.02) before the intervention, *t*(90) = 2.36, *p* = 0.020, *d* = 0.25.

**Fig. 1:**
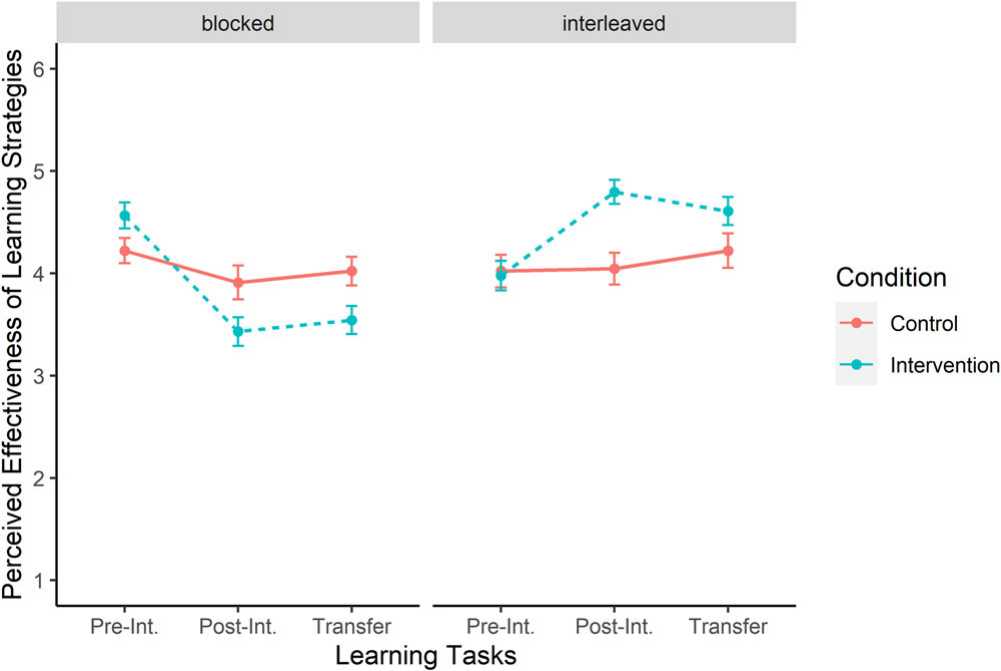
Perceived effectiveness of blocked and interleaved practice across learning tasks. The panel on the left shows the perceived effectiveness of blocked practice, and the panel on the right shows the perceived effectiveness of interleaved practice. Error bars represent the standard error of the mean. Pre-Int. and Post-Int. refer to the pre-intervention and post-intervention tasks. Transfer refers to the delayed-transfer task.

Then, we examined how participants’ learning strategy beliefs changed across time (as reflected by learning tasks) and varied between control and intervention conditions (Fig. 1). Two separate 2 (condition: intervention or control) × 3 (time: pre- intervention, post-intervention, and after a delay) mixed analyses of variance (ANOVA) were conducted on the perceived effectiveness of blocked and interleaved practice, using the afex package^40^ in R.

As for blocked practice, we found a significant main effect of Time, *F*(1.76, 153.11) = 22.65, *p* < 0.001, ${\eta }_{g}^{2}\,$= 0.100, no main effect of Condition, *F*(1,87) = 2.23, *p* = 0.139, ${\eta }_{g}^{2}\,$= 0.015, and a significant Time × Condition interaction, *F*(1.76, 153.11) = 8.20, *p* < 0.001, ${\eta }_{g}^{2}\,$= 0.039. To decompose the interaction, we examined the simple main effect of Time in the intervention and control conditions. In the intervention condition, perceived effectiveness of blocked practice significantly decreased, *F*(2.00, 86.00) = 28.24, *p* < 0.001, ${\eta }_{g}^{2}\,$= 0.229. In the control condition, there was no significant change, *F*(1.54, 67.67) = 2.00, *p* = 0.306, ${\eta }_{g}^{2}\,$= 0.018. Pairwise comparisons (using Bonferroni correction to correct for multiple tests) further revealed that perceived effectiveness of blocked practice was significantly higher in the control condition (*M* = 3.91, *SD* = 1.10) than in the intervention condition (*M* = 3.43, *SD* = 0.95) after the intervention, *t*(87) = 2.19, *p* = 0.031. We observed this difference after the delay as well, control condition: *M* = 4.02, *SD* = 0.94, intervention condition: *M* = 3.54, *SD* = 0.93, *t*(87) = 2.49, *p* = 0.015.

As for interleaved practice, we found a significant main effect of Time, *F*(1.62, 140.7) = 6.44, ${\eta }_{g}^{2}\,$= 0.033, *p* = 0.004, main effect of Condition, *F*(1,87) = 6.29, *p* = .014, ${\eta }_{g}^{2}\,$= 0.038, and a significant Time × Condition interaction, *F*(1.62, 140.7) = 4.32, *p* = 0.022, ${\eta }_{g}^{2}\,$= 0.022. To decompose the interaction, we analyzed the simple main effect of Time in the intervention and control conditions. In the intervention condition, perceived effectiveness of interleaved practice significantly increased, *F*(1.57, 67.44) = 11.62, *p* < 0.001, ${\eta }_{g}^{2}\,$= 0.115. In the control condition, there was no change over Time, *F*(1.64, 72.17) = 0.690, *p* < 0.954, ${\eta }_{g}^{2}\,$= 0.007. Pairwise comparisons (using Bonferroni correction on Alpha levels) further revealed that perceived effectiveness of interleaved practice was significantly higher in the intervention condition (*M* = 4.80, *SD* = 0.80) than in the control condition (*M* = 4.04, *SD* = 1.04) after the intervention, *t*(87) = −3.81, *p* < 0.001. However, this difference was not significant after a delay, Control condition: *M* = 4.22, *SD* = 1.13, intervention condition: *M* = 4.61, *SD* = 0.93, *t*(87) = −1.77, *p* = 0.080.

### RQ2. The use of interleaved practice across time and between conditions

At the pre-intervention task, blocked trials were more frequent than interleaved trials (on average, 0.57 and 0.43, respectively). We examined whether this preference was intentional, by testing whether this frequency was significantly higher than chance (1/6 or 0.16). A one sample t-test revealed that participants’ use of blocked practice cannot be explained by chance alone, *t*(90) = 12.89, *p* < 0.001.

**Fig. 2:**
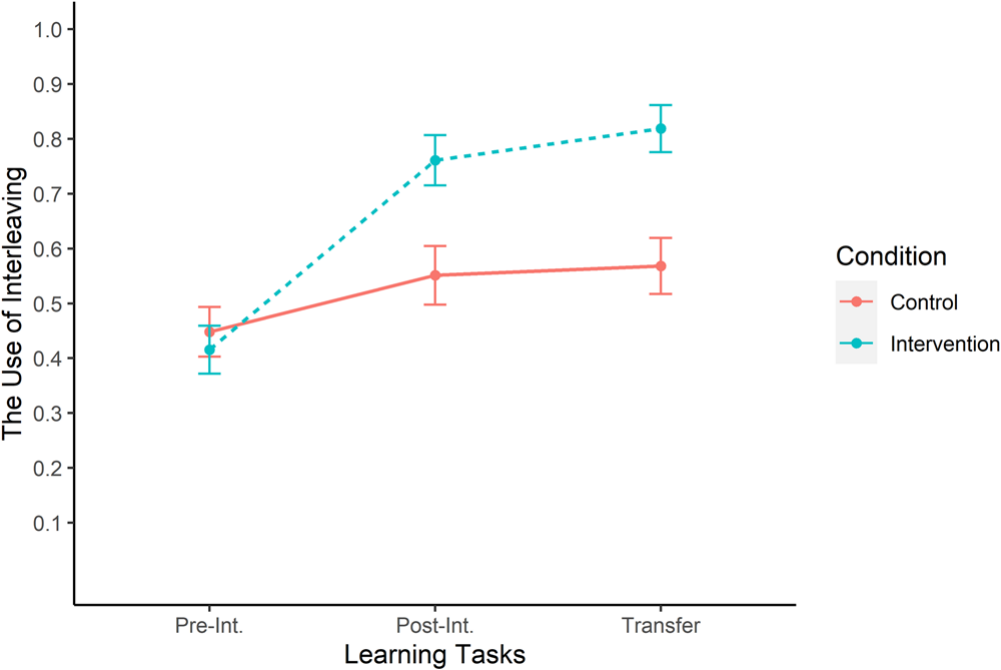
The use of interleaved practice (in proportions) across learning tasks. The change in the use of interleaved practice as a function of the intervention. Error bars represent the standard error of the mean. Pre-Int. and Post-Int. refer to the pre-intervention and post-intervention tasks. Transfer refers to the delayed-transfer task.

Then, we examined how the use of interleaved practice change across time (as reflected by learning tasks) and varied between control and intervention conditions (Fig. 2). A 2 × 3 mixed ANOVA on the use of interleaved practice revealed a significant main effect of Time, *F*(1.50, 133.58) = 30.74, *p* < 0.001, ${\eta }_{g}^{2}\,$= 0.119, main effect of Condition, *F*(1, 89) = 7.48, *p* < 0.001, ${\eta }_{g}^{2}\,$= 0.049, and a significant Time × Condition interaction, *F*(1.50, 133.58) = 9.00, *p* < 0.001, ${\eta }_{g}^{2}\,$= 0.038.

We interpret the main effect of Time in view of the interaction effect. To decompose the interaction, we analyzed the simple main effect of Time in the intervention and control conditions. The results revealed that participants in the intervention condition significantly increased their use of interleaved practice, *F*(1.64, 73.63) = 40.38, *p* < 0.001, ${\eta }_{g}^{2}\,$= 0.266. In the control condition, this increase did not reach significance, *F*(1.38, 60.89) = 2.94, *p* < 0.156, ${\eta }_{g}^{2}\,$= 0.025, although the trend was in the expected direction. Pairwise comparisons (using Bonferroni correction on Alpha levels) further revealed that after the intervention, participants’ use of interleaved practice was significantly higher in the intervention condition (*M* = 0.76, *SD* = 0.31) than in the control condition (*M* = 0.55, *SD* = 0.36), *t*(89) = −2.98, *p* = 0.004). We observed the same difference after a delay as well, intervention condition: *M* = 0.81, *SD* = 0.29, control condition: *M* = 0.57, *SD* = 0.34, *t*(89), *p* < 001.

### RQ3. The influence of interleaved practice on classification accuracy

We examined the association between interleaved practice and classification accuracy, when students followed a learning order organized by an experimenter (strategy implementation during the intervention phase) and when they created their own study order (post-intervention and delayed transfer learning tasks).

#### Experimenter-controlled learning task

Initially, we examined the difference between control and intervention conditions, since the intervention may interact with the classification performance. The results revealed no significant difference between conditions, *t*(88, 88) = −0.68, *p* = 0.499. Then, we examined the influence of learning strategies. A paired sample t-test revealed that interleaved practice (*M* = 6.56, *SD* = 2.97) resulted in better classification performance than blocked practice (*M* = 3.57, *SD* = 2.40), *t*(90) = 10.00, *p* < 0.001, *d* = 1.05, replicating the well-established interleaving effect in learning painting styles.

#### Self-controlled learning tasks

Again, we initially investigated whether the control and intervention conditions differed in terms of classification performance. A 2 × 2 mixed ANOVA revealed no main effect of Time, *F*(1,89) = 2.35, *p* = 0.129, ${\eta }_{g}^{2}\,$= 0.009, no main effect of Condition, *F*(1,89) = 1.51, *p* = 0.222, ${\eta }_{g}^{2}\,$= 0.011, and no Time × Condition interaction, *F*(1,89) = 1.63, *p* = 0.205, ${\eta }_{g}^{2}\,$= 0.006.

Our initial analysis revealed that participants in the intervention condition did not perform better than participants in the control condition, but this analysis did not provide insight on to what extent interleaving rate contributes to accuracy. Note that even those in the control condition interleaved more than 50% of the trials. To estimate the contribution of interleaved practice to the classification accuracy, we constructed two generalized linear mixed-effects models: One for the post-intervention task, and one for the delayed-transfer task. Classification accuracy (correct or incorrect) was the dependent variable, and the use of interleaved practice (in proportion) was specified as a single predictor. We included random intercepts for subjects and items. All analyses were carried out, using the lme4 (vers. 1.1-32)^41^ and lmerTest packages (vers. 3.1-3)^42^.

The results revealed that the use of interleaved practice was associated with higher classification accuracy when students learned painting styles (post intervention task), Estimate = 1.56, *SE* = 0.54, *z* = 2.77, *p* < 0.005. More specifically, every single switch between the painting styles of different artists increased the odds of correct classification by 4%. A similar correlational pattern was observed when participants studied bird species (delayed-transfer task), Estimate = 1.65, *SE* = 0.47, *z* = 3.48, *p* < 0.001. Here, every *single switch* between the images of different bird species increased the odds of correct classification by 5%. For interested readers, we provided bi-variate correlations between classification performance and switch rates in supplementary note 1.

### Exploratory analyses

How did students engage in interleaved practice? To answer this question, we examined another measure of interleaved practice: The longest run (i.e., length) of an interleaved sequence. This measure captures the maximum number of consecutive times a participant switched between categories across a set of exemplars. For example, in a sequence of ABCCBA, there are four switches (ABC, CBA). However, the length of this interleaved sequence is two, since there are two switches before (and after) the run is interrupted from C to C. We investigated how the length measure differed as a function of our intervention and how it was associated with classification performance.

The results revealed a significant difference between the control and intervention conditions in terms of how participants engaged in interleaved practice. At the post-intervention task, the length of the interleaved sequence was higher in the intervention condition (*M* = 21.15; *SD* = 13.96) than in the control condition (*M* = 13.67, *SD* = 13.93), *t*(88.97) = −2.56, *p* = 0.012. We observed the same pattern in the delayed-transfer task, intervention condition: *M* = 24.67, *SD* = 13.32; control condition: *M* = 13.37; *SD* = 13.35, *t(*88.95) = −4.04, *p* < 0.001. Finally, we examined the bivariate correlation between participants’ longest interleaving sequence and their classification accuracy. We found a significant association both at the post-intervention task, *r*(89) = 0.22, *p* = 0.035, and at the delayed-transfer task, *r*(89) = 0.33, *p* < 001.

## Discussion

The present study aimed to improve students’ self-regulated use of interleaved practice for category learning tasks in authentic situations, in which students are required to create their own study order at the level of exemplars. To this end, we tested the efficacy of a learning strategy intervention that combined refutation-based instructions and visual metacognitive prompts, which previously showed positive effects at the strategy level^30^. The results revealed a strong intervention effect: Students corrected their erroneous beliefs, improved their use of interleaved practice, and further implemented this strategy in a near-transfer task, which took place after a delay (five to seven days). Importantly, interleaved practice was associated with better classification accuracy, highlighting the importance of such strategy interventions.

Several studies have shown that students seldom engage in interleaved practice^9,10^ and believe that blocked practice leads to better learning^27,28^. Supporting our first and second hypotheses, we replicated this unfavorable position of interleaved practice: Before the intervention phase, perceived effectiveness of blocked practice was higher than of interleaved practice, and students engaged in blocked practice more frequently than interleaved practice. Interestingly, students’ initial beliefs and engagement in interleaved practice was higher than in previous studies. For example, Tauber et al.^21^ found a stronger preference for blocked practice: In their study, students blocked 90% of the trials (except 70% in Exp. 4), against 55% in our study. This discrepancy may result from the interaction between the number of to-be-learned exemplars and categories^38^. In our study, students learned six categories, studying six exemplars for each category. Tauber et al.^21^ asked students to learn eight and 12 categories, studying six exemplars for each category. Arguably, students might engage in blocked practice more often when task demands are increasing. Alternatively, it is plausible that students are gaining some awareness toward desirably difficult strategies. Indeed, recent studies challenge the presumption that students have no metacognitive knowledge about desirable difficulties^10,43^.

Confirming our second hypothesis, students interleaved more frequently across time, and this improvement was larger for the intervention condition. Students in this condition interleaved about 80% of the trials at the post-intervention task and delayed (near-transfer) learning task. Crucially, we observed this improvement in a learning task that required students to create their own study order. Although previous intervention studies revealed promising results^18^, these interventions took place in experimenter-controlled learning tasks, where students chose which strategy to implement as opposed to arrange the exemplars by themselves. Evidently, this prior research has difficulties in capturing the complexity of authentic learning situations, in which students make many small decisions to organize their learning materials instead of making one overall decision to (not) use interleaving. Such situations allow for more variance in terms of students’ engagement in blocked and interleaved practice and impose higher demands on students with regard to the mental resources that should be devoted to metacognitive monitoring and control of learning^44^. Yet, we have demonstrated that students can enhance their use of interleaved practice in these resource-demanding situations, with the help of refutations and experience-based metacognitive prompts.

Several factors might have contributed to the effectiveness of our strategy intervention. Here, we will discuss three main factors. First, it is plausible that refutations played an important role in helping students to develop accurate knowledge about learning strategies. With refutations, we did not provide students with normative information alone but also challenged their existing beliefs^31,32^. This confrontation might have paved the way for creating dissatisfaction with existing concepts^34^ and study behavior, which is an essential first step for changing towards more effective strategies. However, it is worth mentioning that previously used instructions^19^ also shared some key characteristics of refutations – although these instructions were not explicitly described as refutations. In this study, we might have increased the persuasive power of refutations with our design choices, through explanations^45^, credible sources^46^, and its timing^36^. Note that we expanded the content of refutations to a) the way that blocked and interleaved practice foster learning, and b) how task experiences influence study decisions. Moreover, we provided students with this information upfront – before they could reinforce their erroneous beliefs with misleading on-task experiences. Arguably, this approach might be more convincing than previously-used instructions that informed students about the efficacy of learning strategies alone or after strategy implementation. Finally, metacognitive prompts might have helped students to recognize that interleaved practice actually works for them. Direct experience (i.e., using blocked and interleaved practice) is a powerful catalyst for internalizing theoretical information, but it is a challenging task for students to monitor their learning progress continuously, while performing a learning task, and associate this information with the effectiveness of learning strategies^1^. Our metacognitive prompts (both the questions that elicit monitoring judgments and the visual summaries) might have eased this mental burden on students and contributed to the internalization of theoretical information – although prior research found no direct benefits of metacognitive prompts, indirect benefits are reported (e.g., increased confidence in one’s decision).

Together, our findings provide further support that theory-based methods (e.g., refutations) can be reinforced with experience-based methods (e.g., metacognitive prompts) rather than performance feedback – i.e., providing students with test scores (Your score from Strategy A is X while from Strategy B is Y). In our view, a drawback of performance feedback lies in its strong focus on learning outcomes, which may potentially contradict with normative information and reinforce students’ pre-existing erroneous beliefs. For instance, when performance feedback is derived from immediate test scores, some students may obtain higher results with blocked practice, perform equally well, or obtain only a ‘small’ benefit of interleaved practice^47^. In such situations, performance feedback might fail to improve the use of interleaved practice^18^ or have unintended negative consequences than positive outcomes, such as by reinforcing pre-existing erroneous beliefs^48^.

Confirming our third hypothesis, we found that interleaved practice was associated with better classification accuracy, both at the post-intervention and delayed-transfer learning tasks. In general, this finding supports the proposition that the interleaving effect is not bounded to experimenter-controlled learning tasks; students also benefit from interleaving when they organize learning materials themselves^37,38^. Yet, we should acknowledge that several studies could not confirm this proposition or found blocking effect in self-controlled learning tasks^20,38^. One possible explanation is self-controlled learning tasks might overload the working memory capacity of students due to mental resources that need to be devoted to metacognitive monitoring and control of learning^44^. With each choice, students need to decide whether a category requires further study or identify the next appropriate category to focus on. This secondary load^39^ might compete for the limited mental resources that would have been otherwise devoted to learning processes that benefit category learning (e.g., contrasting categories). It is plausible that we found an interleaving effect because our strategy intervention took this load off of students.

A limitation of this study is that we considered interleaved practice a desirable difficulty in a context where students learned visual categories^49^. However, whether a strategy is desirable depends on the task demands. We should, therefore, acknowledge that students may benefit from blocked practice in certain conditions, depending on the complexity of learning tasks^50^, (dis)similarity of to-be-learned materials^51,52^, and test type^53^. Second, in the interleaved practice literature, it is common to rely on learning tasks with relatively low authenticity, such as learning painting styles and bird species. Therefore, it remains unclear whether students would adopt a similar approach in their actual study behavior, when learning materials become more relevant and important for them, when other factors may play an important role, such as motivation (e.g., task-interest, learning goals: mastery versus performance) and prior knowledge. An important next step is to examine whether this intervention translates to real-world situations, such as to classroom settings, and if there is an optimal extent of interleaving.

In conclusion, the present study showed that with the help of a strategy intervention that combines refutations and metacognitive prompts, students can overcome their erroneous beliefs about learning strategies and use more effective strategies in a more authentic learning setting. The findings indicated that our intervention is highly effective; it allows students to develop accurate knowledge about learning strategies and to prove that effective strategies actually work for them. Future research should examine the efficacy of this approach in classrooms and real-life settings.

## Methods

### Transparency and openness statement

This study’s research questions, hypotheses, and data analyses plan were pre-registered. Data, custom codes, and materials are publicly available on Open Science Framework (OSF).

### Design and participants

We used a 3 × 2 mixed-subjects design. Participants performed three learning tasks within the context of category learning with visual materials: Pre-intervention task, post-intervention task, and delayed-transfer task. The intervention was manipulated between subjects (intervention: yes or no). In the pre- and post-intervention tasks, participants learned the painting styles of various artists. In the transfer task, they learned about bird species.

We determined the required sample size based on the expected impact of refutations on misconceptions: To adopt interleaved practice, students should correct their pre-existing beliefs about blocked and interleaved practice. Furthermore, refutations constituted the necessary and sufficient component of our intervention. A recent meta-analysis by Schroeder and Kucera^54^ revealed a medium-sized effect (*g* = 0.41) of refutations on misconceptions. We conducted an a priori power analysis for each main effect and interaction using G*Power software^55^. Alpha was set at 0.05, power was set at 0.80, and correlation among repeated measures was kept default (0.5). Based on this calculation, 84 participants would be sufficient.

As we expected a 15% drop-out rate, we recruited 96 participants ( ~ 20 years of age, 52 female, 42 male, 2 non-binary). Participants were first, second, and third-year undergraduate students at Maastricht University, the Netherlands. Five participants did not return to the second study session within the time-period, indicated in our pre-registration form. Those participants were omitted from data analyses. Additionally, two participants provided incomplete data on Day 1 (on one measure) due to experimenter error. Because these participants completed the majority of the study, we removed these participants only from the respective analyses. Each participant was rewarded with a 15€ voucher for their participation. The ethical committee of Faculty of Health, Medicine, and Life Sciences at Maastricht University, the Netherlands, approved the study: REC/2022/091.

### Stimuli and learning tasks

Our stimuli consisted of 180 paintings and 48 bird images, which belong to 24 artists and six bird species, respectively. The stimuli were divided into four subsets, depending on the phase that participants completed: pre-intervention task, post-intervention task, delayed-transfer task, and the intervention. Pre-intervention task contained 36 paintings, six paintings by each of six artists, obtained from Khan and colleagues^56^. Post-intervention task contained 48 paintings, eight paintings by each of six artists (six paintings for studying and two paintings for assessment), previously used by Sun and colleagues^18^. Delayed-transfer task contained 48 bird images, eight images by each of six bird species (six images for studying and two images for assessment), previously used by the authors. The remaining 96 paintings, eight paintings by each of 12 artists (six paintings for studying and two for assessment) were used during the intervention. These paintings were previously used by Kornell and Bjork^11^.

### Intervention

The intervention consisted of three main components: refutations, strategy implementation, and visual metacognitive prompts (the authors, under review). Regarding refutations, two instructional texts were created (see Supplementary Figs. 1–2). The first text challenged the belief that blocked practice leads to better learning than interleaved practice. The second text challenged the inaccurate monitoring of effort and learning. Both texts shared the same characteristics. First, erroneous beliefs were clearly described to students (e.g., “Many students believe that blocked practice leads to better learning than interleaved practice”). Then, we explicitly stated the incorrectness of erroneous beliefs (e.g., “However, this belief is false”). Finally, participants were provided with the correct information (e.g., “Interleaved practice leads to better learning than blocked practice”) and evidence-based explanation (e.g., “Interleaved practice highlights the differences between categories”). Where necessary, we provided citations and added graphics to support the textual information. The first refutation included 273 words and the second refutation included 278 words. These materials can also be found in OSF link.

The strategy implementation gave students an opportunity to experience the difference between blocked and interleaved practice. Students performed an experimenter-controlled learning task, in which they studied the painting styles of 12 artists (six with blocked practice and six with interleaved practice, counterbalanced) and monitored their on-task experiences (i.e., perceived effort and learning) across time. Half of the paintings were studied through blocked practice, and the other half was studied through interleaved practice. Blocked units contained six paintings by one artist. Interleaved units contained six paintings, one painting by each of six artists. A fixation-cross preceded all paintings and remained on the screen for one second. Paintings were shown one at a time at the center of the screen for three seconds, with the last name of the artist above. Participants studied the units in the following order: B-I-I-B-B-I-I-B-B-I-I-B^11^. At the end of each unit, participants were asked to rate their perceived effort (i.e., how much mental effort does this strategy require?) and perceived learning (i.e., how likely do you think you will be able to remember the painting/paintings of this/these artist/s?) on a 9-point, one item Likert scale.

**Fig. 3:**
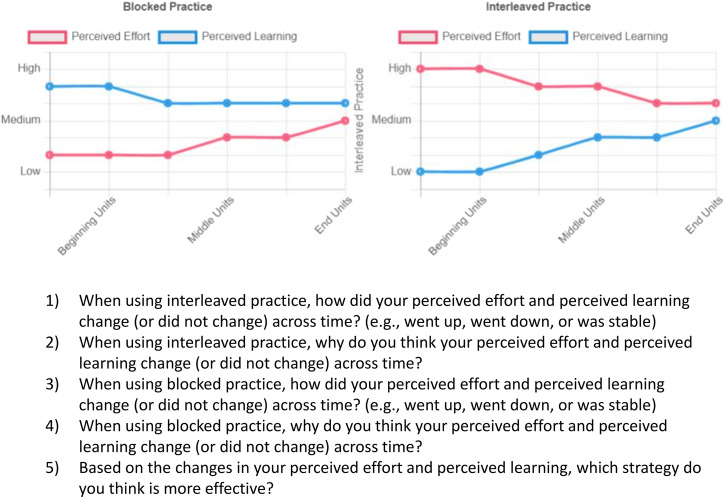
Visual metacognitive prompts (an example from a participant). Perceived effort and perceived learning associated with blocked practice was shown on the left panel. Perceived effort and perceived learning associated with interleaved practice was shown on the right panel. The prompt questions were presented to students below the visual summaries.

On visual metacognitive prompts (Fig. 3), students could examine the development of their on-task experiences with blocked and interleaved practice. To foster comparison between learning strategies for learners, we grouped the experiences by strategy. That is, two line graphs were displayed to students. On the left graph, students could examine how their on-task experiences of perceived effort and perceived learning with blocked practice changed across time. On the right graph, students could examine how these on-task experiences with interleaved practice changed across time. Underneath the line-graphs, prompt questions were posed to students. These prompt questions aimed that participants a) focused on the temporal changes in their on-task experiences, b) interpreted those temporal changes (or no changes), and c) created associations between those changes and the efficacy of learning strategies.

### Learning strategy beliefs

Participants rated the perceived effectiveness of blocked and interleaved practice for long-term learning on two separate single-item Likert-type scales (ranging from 1 = not at all effective to 6 = extremely effective). We complemented these single-item scales with an open question, in which students were asked to explain why they considered blocked and interleaved practice (in)effective.

### Coding of study sequences

On the pre-intervention, post-intervention, and delayed-transfer tasks, participants could create their own study order, by making free choices. Each task required participants to make 35 unique choices to study all materials. We operationalized interleaved practice following Lu et al.^38^. That is, for each choice, participants could decide whether they wanted to ‘stay’ within a category or ‘switch’ between categories. Switch decisions (when category n is different from category n-1) were counted as an instance of interleaving. For each participant, we calculated the proportion of switches (n_switch / 35).

### Classification accuracy

We measured participants’ ability to classify novel exemplars (paintings and bird images) to appropriate categories (artists and bird species). We aimed to assess the influence of both self-organized study sequences and the experimenter-controlled study sequence. The former was measured after the post-intervention and delayed-transfer learning tasks. These classification tests were composed of 12 trials, two novel exemplars per category. The latter was measured after the strategy implementation. This test was composed of 24 trials, two novel exemplars per category. During the trials, exemplars were shown one at a time at the center of the screen, with the names of the categories below. Participants were asked to select the appropriate category with no time restriction.

### Procedure

**Fig. 4:**
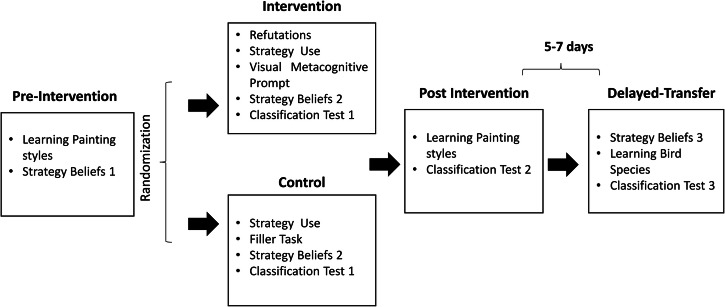
Procedure. A visual overview of the study procedure.

Throughout the study (Fig. 4), participants engaged in four different phases: Pre-intervention → Intervention → Post-intervention → Delayed-transfer. The first three phases were completed on the same session at a computer lab. The last phase was completed online, between five and seven days after the first session. During the pre-intervention, post-intervention, and delayed-transfer tasks, participants could create their own study order. These tasks were programmed in SoSci Survey platform. The intervention was delivered through Qualtrics. Demographic information and informed consents were collected before the pre-intervention phase.

**Fig. 5:**
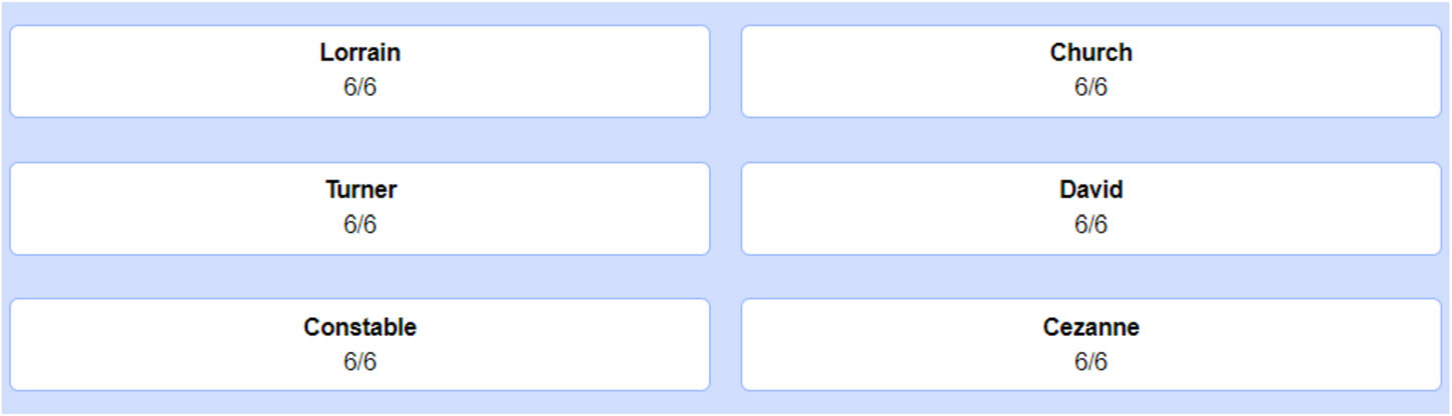
Selection page. Using this selection tool, students created their own study order. They clicked on the buttons to view an exemplar. Below the buttons, they could also see the remaining number of exemplars for study.

The pre-intervention task provides a baseline for how students typically arrange visual exemplars in a self-study. Thus, prior to this task, participants were not informed about blocked and interleaved practice, as well as their efficacy in category learning. As for a general explanation, we told participants that they will learn the painting styles of various artists, and they should study these paintings in the order they want, but in a way that would maximize their learning. In the end of this brief instruction, we explained participants how to use the selection page (Fig. 5). On this page, there were six buttons, displayed to participants in two columns. All buttons contained two elements: The last name of an artist and the remaining number of paintings. To open a painting, participants clicked on the buttons. For example, if they wanted to study a painting of Lorrain, they clicked on Lorrain. Then, a painting of Lorrain appeared on the screen for four seconds. Then, they returned to the selection page again. A button turned inactive once participants studied all the paintings of an artist. Following this brief explanation, participants performed the pre-intervention task and continued to the intervention phase.

In the beginning of the intervention phase, we explained participants how exemplars can be organized in blocked and interleaved fashion. Note that this introduction did not reveal the efficacy of learning strategies. After this explanation, participants indicated their perceived effectiveness of blocked and interleaved practice for the first time and were randomized into intervention and control conditions. Those in the intervention condition (*n* = 46) read the refutations first, before they tried out blocked and interleaved practice. Those in the control condition (*n* = 45) did not read the refutations but only tried out strategies. After this strategy use, participants in the intervention condition were provided with visual metacognitive prompts. Meanwhile, the others read an article about the Internet ( ~ 1000 words)^57^ as a filler task. Subsequently, all participants reported their perceived effectiveness of blocked and interleaved practice for the second time and took part in a classification test.

Next, the post-intervention task took place. Here, participants learned the painting styles of novel artists, which was followed by a distractor task and a classification test. As for distraction, they solved five simple equations (e.g., 4x + 9 = 45, what is x?). The first session was over once participants answered all the questions in the classification test. In the end of this session, we reminded participants that second session will be made available five days later, and they should complete this session within two days.

The procedure for the delayed transfer phase was similar to those of post-intervention phase. One difference was that participants rated the perceived effectiveness of blocked and interleaved practice before they performed the learning task. Second, instead of learning painting styles, participants learned to identify bird species. The remaining procedure was identical to the post-intervention phase. Upon completion of the study, participants were rewarded with 15 € vouchers.

### Reporting summary

Further information on research design is available in the Nature Research Reporting Summary linked to this article.

### Supplementary information

Supplemental Information

Reporting summary

## Data Availability

All data and materials are available at OSF: https://osf.io/6vm7z/?view_only=d9caf564f48b4c4f8166b3c2e38c1cf4.

## Code availability

Custom codes are available at OSF: https://osf.io/6vm7z/?view_only=d9caf564f48b4c4f8166b3c2e38c1cf 4.
