# Supplementary material for: Optimizing self-organized study orders: combining refutations and metacognitive prompts improves the use of interleaved practice
Source: NPJ Sci Learn. 2024 Apr 24;9:33. doi: 10.1038/s41539-024-00245-7 (PMC11043372; doi:10.1038/s41539-024-00245-7)
Supplement: Supplementary file 1 — Supplemental Information [file 41539_2024_245_MOESM1_ESM.pdf]

Supplementary Fig. 1. Refutations on the effectiveness of blocked and interleaved practice

Before we begin with the study, we would like to inform you about two common misunderstandings, or 'misconceptions' about learning, which might undermine students' educational achievements.

{Page break}

The first misconception concerns the effectiveness of blocked and interleaved practice: Many students believe blocked practice leads to better learning (i.e., better memory, better test results) than interleaved practice does. However, **this belief is false**. Research from the last decades shows that **interleaved practice is in fact a more effective strategy** to learn study materials than blocked practice. This applies to multiple study materials (Brunmair & Richter, 2019). For example, interleaved practice leads to better educational achievements when learning visual patterns (e.g., learning to diagnose diseases on x-rays).

Why is interleaved practice more effective than blocked practice for learning? Let's think about our zoology student. In a later test, this student needs to identify butterfly families from novel pictures. To success in this task, it is important that our student can **recognize the features that differ between the butterfly species** such as the differences in the shape of the wings between a Baltimore and Tiger butterfly (See Picture below).

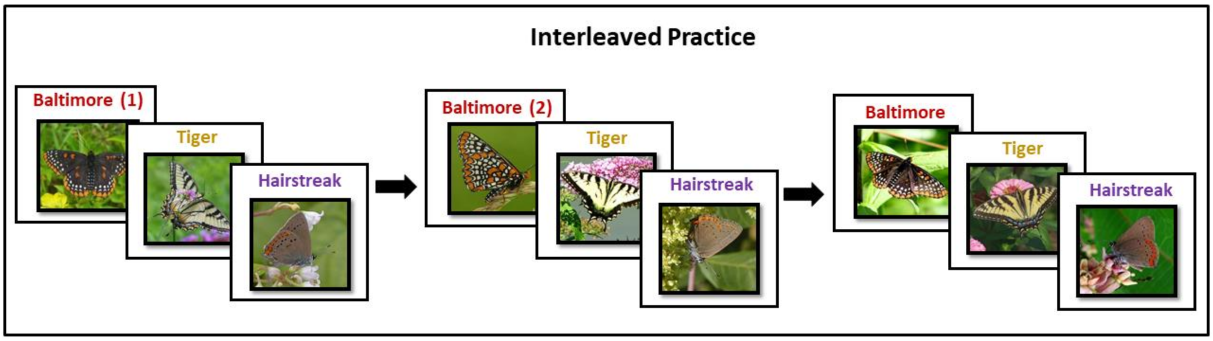

Interleaved practice leads to better learning because it **highlights those differences**. Seeing those differences, in turn, improves our ability to recognize each species later in a different context (Kang & Pahsler, 2012). Another reason that interleaved practice improves learning is because it **creates a time interval** between the examples of the same species. As indicated in the figure above, when studying Baltimore again, we **recall information** about the Baltimore species from our memory. This recall attempt, in turn, fosters our memory and slows down forgetting (Foster et al., 2016). In contrast to these advantages**, in blocked practice**, you only **see the similarities within the species**. This lower the ability to recognize each species later.

Supplementary Fig 2. Refutations on the inaccurate monitoring of effort and learning

The second misconception many individuals hold concerns how we interpret situations when learning costs much effort. Often, we are inclined to believe that we learn more when learning feels easy. Therefore, we often choose the learning strategy that makes learning feel easier. However, **this interpretation is false**. Instead, like many desirable behaviors (eating healthy, exercising), using effective learning strategies **cost effort, does not feel easy, but pays off in the long term** (Bjork & Bjork, 2011). This also applies to interleaved practice: it often feels not easy, but actually leads to better learning than blocked practice. In what follows, we will explain this misinterpretation using the results of a recent study.

In this study, students used blocked and interleaved practice to learn visual patterns. While using these strategies, students also rated how much effort it took them (i.e., perceived effort) and how much they thought they learned (i.e., perceived learning). Finally, they took a test **after 10 minutes**

{Page break}

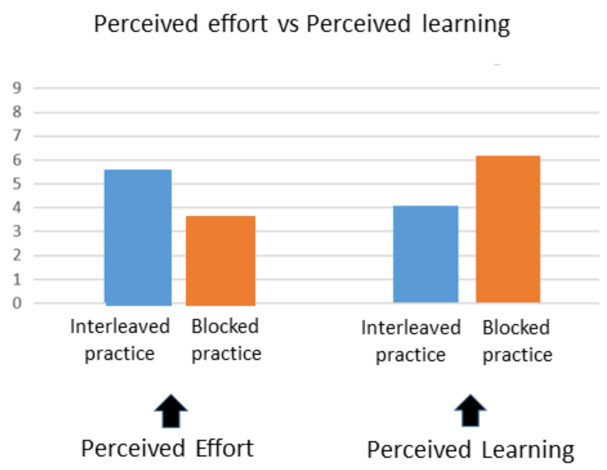

As you can see in the graph, **interleaved practice** cost students **more effort** than **blocked practice**. At the same time, students thought that they learned more with **blocked practice than** with **interleaved practice.** But remember, like exercising, you may not immediately observe the improvements in your learning, especially when you are trying harder.

{Page break}

The next figure shows how their test scores (actual learning) differed from their perceived learning.

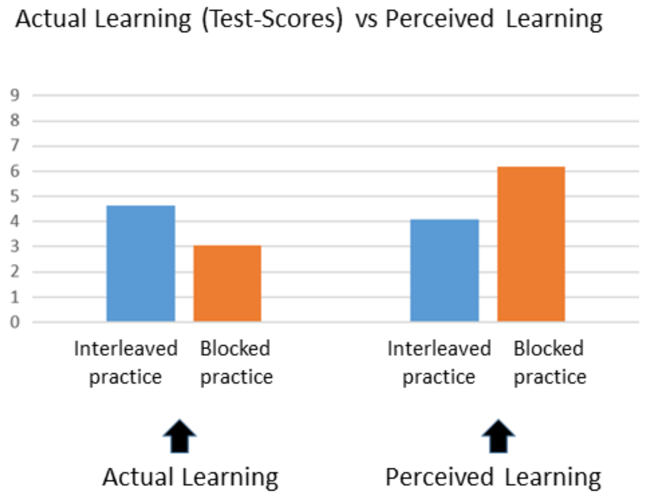

As you can see in the figure, **interleaved practice** led to higher test scores than **blocked practice**, **although the students felt the opposite**. This study shows that interleaved practice is a more effective learning strategy, even though students **feel** it is more effortful and leads to less learning. In summary, how we think we learn best differs from how we actually learn best. This also applies to **interleaved practice**, which is **more effortful but also more effective** than **blocked practice**

Supplementary Note 1. Bivariate correlations between the switch rates and performance

As for the post-intervention task, we found a significant and positive correlation between the interleaving rate and classification accuracy, r (89) = .27, p = .009. This correlation was .35 for the delayed-transfer task, p < .001.
